# Microwave-hydrothermally synthesized Ru_x_NiCo_2-x_O_4_ spinel oxide nanoparticles for high-performance pseudocapacitor electrodes

**DOI:** 10.1038/s41598-026-52710-5

**Published:** 2026-05-12

**Authors:** I. Tejaswi, K. Anusha, S. Katlakunta, Ch. Shilpa Chakra, N. Venkata Prasad, Md. Shareefuddin, D. Sreenivasu, B. Ravinder Reddy

**Affiliations:** 1 Department of Physics, University College of Science, Osmania University, Hyderabad, India; 2 Nano-Energy R&D Lab, Centre for Nano Science and Technology, UCESTH, Jawaharlal Nehru Technological University, Hyderabad, India

**Keywords:** Ru_x_NiCo_2-x_O_4_ spinel oxides, Microwave hydrothermal method, Pseudocapacitive behavior, Transition metal oxides, Electrochemical energy storage, Supercapacitor electrodes, Chemistry, Energy science and technology, Materials science, Nanoscience and technology

## Abstract

A series of Ru_x_NiCo_2-x_O_4_ (RNCO; x = 0.00–0.10) spinel oxides at nanoscale were synthesized using a microwave hydrothermal (M-H) process. Structural, morphological, and electrochemical characteristics were investigated by various characterizations. The X-ray diffraction results confirm the formation of a single-phase cubic spinel structure with an *Fd*-3 *m* space group for all compositions. The lattice parameter decreases linearly from 8.089 Å (x = 0.00) to 8.085 Å (x = 0.10). Field emission scanning microscopy images reveal nanoparticles with elongated and spherical morphologies, with average grain sizes ranging from 32 nm (x = 0.06) to 55 nm (x = 0.10). Fourier transform infrared spectra exhibit characteristic metal–oxygen vibrational bands around 656 cm^−1^, confirming spinel oxide formation. X-ray photoelectron spectroscopy analysis verifies the presence of mixed valence states of Ni^2+^/Ni^3+^, Co^2+^/Co^3+^, and Ru^3+^/Ru^2+^. Electrochemical studies demonstrate enhanced pseudocapacitive behavior with Ru incorporation. The results were corroborated to Randles’s, Dunn’s and impedance analysis. Notably, the Ru_0.06_NiCo_1.4_O_4_ (RNCO-4) electrode delivers a superior specific capacitance of 1541 F g^−1^, and an energy density of 53.35 Wh kg^−1^ at a power density of 2233 W kg^−1^. These results indicate that Ru-substituted NiCo_2_O_4_ spinel oxides synthesized via the Microwave-Hydrothermal (M-H) route resulted in novel electrode materials for supercapacitor applications.

**Supplementary Information:**

The online version contains supplementary material available at 10.1038/s41598-026-52710-5.

## Introduction

Supercapacitors offer higher work performing rates, enhanced energy densities than conventional energy storage devices like capacitors and batteries^1,2^. Supercapacitors (SCs) synonymously called as electrochemical capacitors (ECs) that commercially created significant interest^3^. They have been probed for enriched properties that include higher rate of charge/discharge, high power density, and large working span with ecofriendly safety^4,5^. These are replacing power sources in rechargeable batteries and fuel cells. The ECs find their place in pacemakers, power storage, air filled sachets, and electrical vehicles^6^. The electrode materials decide the performance of ECs. Currently three main types of electrode materials used in SCs, which are carbon materials, conductive polymers and metal oxides^7^. Global urge is to increase the energy storage capacity of electrically driven batteries and were examined NiCo_2_O_4_ exhaustively to enhance the energy storage capacity of Li-ion batteries^8^. A decrease in charge transfer barrier, and having more accessible active sites leads to an increased oxygen vacancy concentration in Ru-based NiCo_2_O_4_ materials. Therefore, these material exhibits excellent oxygen evolution reaction (OER) activity with a lower overpotential (< 230 mV) and reach the current density of 10 mA/cm^2^. In addition, Ru-NiCo_2_O_4_ have shown superior hydrogen evolution reaction (HER) activity, compared to the pristine based NiCo_2_O_4_^8–10^. On the other hand, Spinel oxide processes unoccupied sites result in the formation of large magnitudes of interstitial positions which favor the cation migration thereby modifying the valency of transition metals^11^. Initially, many cations with low activation energies move swiftly, which lowers the resistance and inertness^12^. Secondly, a wide variety of metal oxides exhibit rich structural diversity, which offers opportunities to tailor their physical and chemical properties, including capacitance^13^. However, nickel cobaltite-based electrode materials still face several challenges in meeting the advanced performance requirements of next-generation energy storage devices. Therefore, the rational design of novel micro or nanostructured electrode materials is imperative for further development of electro-chemical SCs^14–18^. Previous studies indicated that ternary transition metal oxides have attracted considerable attention in supercapacitor research because of their high conductivity and electrochemical activity^19^. Metal oxides in triply transition state such as ZnCo_2_O_4_^20,21^. CuCo_2_O_4_^22,23^, MnCo_2_O_4_^24,25^ have been probed to obtain high specific capacitance, especially in NiCo_2_O_4_^26,27^, The specific capacitance (C_p_) of NiCo_2_O_4_ core mixtures used for electrode was found to be 597 Fg^−1^ at 1 Ag^−1^^28^. NiCo_2_O_4_ nanocubes as electrode have shown the C_p_ of 795.6 Fg^−1^ at Ag^−1^^29^. It is found that NiCo_2_O_4_ / RGO hybrid composites is around 947 Fg^−1^ at 0.5 Ag^−1^ and Ni(OH)_2_ and Co–Ni(OH)_2_ were about 1038 Fg^−1^^30^. The Al_x_Ni_1-x_Co_2_O_4,_ of about 356 Fg^−1^ at 1 Ag^−1^^31^. Porous nickel cobaltite nanosheets deposited on nickel foam has C_p_ of 795 Fg^−1^ at Ag^−1^ and NiCu_x_Co_2-x_O_4_ has got 250.66 Fg^−1^ at 1 Ag^−1^^23^, ^32^. Mesoporous NiCo_2_O_4_ nanoscale pointed edges were grown on 3D graphene-nickel lather showed about 1588 Fg^−1^ at 1 Ag^−1^^33^. Ni_1.5_Co_1.5_O_4_ exhibited 502.8Fg^−1^ at 1 Ag^−1^^34^ and Core shell N-doped active carbon fiber-graphene showed 552.8 Fg^−1^ at 0.1 Ag^−1^^35^. The ultrathin mesoporous RuCo_2_O_4_ nano flakes exhibited 1469 Fg^−1^ at 6 Ag^−1^^36^. Among various transition metal oxides, platinum group element Ru has been emphasized due to its large specific capacitance, fast back redox reaction rates, thermal invariance, and metallic natured conduction, over a regular repetition with large capacbility^37^. Recent research studies on hydrothermally synthesizing a bimetallic cobalt vanadium oxide have shown superior bifunctional supercapacitor and electrolysis applications^38–41^.

In view of aforementioned importance of OER and HER performance, Ru based NiCo_2_O_4_ materials are being prepared in many methods. Ru based NiCo_2_O_4_ materials has both electrode (anode and cathode) catalysts in KOH solution. Keeping in view of the aforementioned importance of spinal-oxide nanomaterials with a traditional electrode are being designed globally in order to enhance the supercapacitance. The electrode, especially, Ru improves the limitations such as conductivity and stability cycling. It is a known fact that the nickel cobaltite enhances the ionic transport and electronic conductivity in the spinal structure. In this work, Ru was substituted into the NiCo_2_O_4_ lattice in order to leverage the conductivity and finally brings the cost-effectiveness, compared to other single metal oxide-based materials.

## Results and discussions

**Fig. 1:**
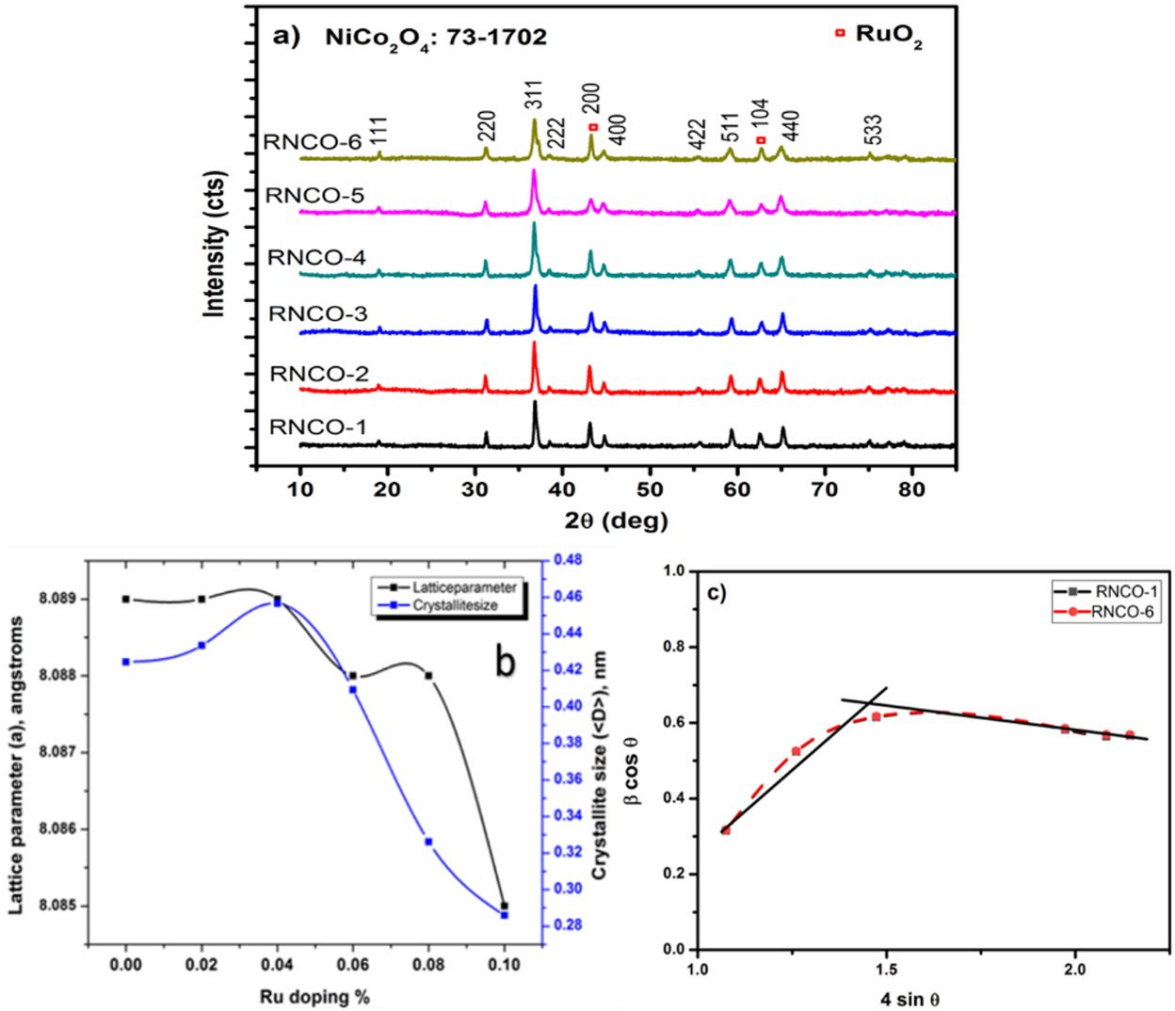
(**a**) The XRD pattern of RNCO, (**b**) Ru doping% vs Lattice parameter and Crystallite size (x10), (**c**) W-H plot.

The crystal structure of NiCo_2_O_4_ and Ruthenium doped NiCo_2_O_4_ (RNCO) were examined by XRD depicted in Fig. 1a. The peaks at 18.89°, 31.2°, 36.72°, 38.5°, 43.24°, 44.7°, 55.55°, 59.15°, 62.72°, 64.95° and 75.23° are corresponding to the crystal planes of (111), (220), (311), (222), (200), (400), (422), (511), (104), (440) and (533) respectively which are matched with the JCPDS card no 20–781. Two minor peaks related to RuO_2_ phases were observed at 43.24° and 62.72° for higher concentrations of Ru^35,36^. The lattice constant (*a*), crystallite size (< *D* >), X-ray density (ρ) and Dislocation density (δ) have been calculated using the following formulas:

1 $$a=\frac{d}{\surd ({h}^{2}+{k}^{2}+{l}^{2})}$$

where *d* is the interplanar distance and *h*, *k*, *l* are miller indices.

The average crystallite size is calculated using the Scherer Equation,

2 $$\langle D\rangle = \frac{k\lambda }{\beta \mathrm{c}\mathrm{o}\mathrm{s}\theta }$$

where *k* is Scherer constant or shape factor; *λ* is X-ray wavelength; *θ*: Bragg angle and *β*: Full Width at Half Maximum (FWHM) of the diffraction peak, measured in radians.

The Dislocation Density (*δ*):

3 $$\delta =\frac{1}{{\langle D\rangle }^{2}}$$

where < *D* > is the crystallite size in nm. The X-ray density (*ρ*):

4 $$\rho = \frac{ZM}{{Na}^{3}}$$

where *Z* = 8 (for spinel structure), *M* is the molecular weight, *N* is Avogadro’s number, and *a* is the lattice parameter in Å.

Table 1 shows the regular repeating arrangement of atoms/ions defined as lattice stretch (*a*), mean crystalline dimension (D), X-ray density (*ρ*) and dislocation density 1/ < D > ^2^ of Ru_x_NiCo_2-x_O_4_ (*x* = 0, 0.02, 0.04, 0.06, 0.08, and 0.10) samples. The sintered powders were labeled as RNCO-1, RNCO-2, RNCO-3, RNCO-4, RNCO-5, and RNCO-6 respectively. The crystal facet of the genuine NiCo_2_O_4_ specimens and those doped with Ruthenium (RNCO) were examined by XRD, depicted in Fig. 1a. The peaks of 2θ values observed at 18.89°, 31.2°, 36.72°, 38.5°, 43.24°, 44.7°, 55.55°, 59.15°, 62.72°, 64.95° and 75.23° are corresponding to the crystal planes of (111), (220), (311), (222), (200), (400), (422), (511), (104), (440) and (533) respectively, which are in concurrent matched with the JCPDS card no 20–781. The (311) peak of RNCO-4 has highest intensity.

**Table 1:** Physical parameters of RNCO crystalline material.

Sl. No	Ru doping (x)	Lattice parameter a (Å)	Crystal size < D > nm	X-ray density X 10^–24^ (gcm^−3^)	Dislocation density 1/ < D > ^2^
1	RCNO-1 (x = 0)	8.089	4.246	5.83	5.545
2	RCNO-2 (x = 0.02)	8.089	4.336	5.85	5.318
3	RCNO-3 (x = 0.04)	8.089	4.566	5.88	4.795
4	RCNO-4 (x = 0.06)	8.088	4.093	5.89	5.967
5	RCNO-5 (x = 0.08)	8.088	3.261	5.90	9.398
6	RCNO-6 (x = 0.10)	8.085	2.859	5.94	12.226

Two characteristic peaks related to RuO_2_ phases found at 43.24° and 62.72° in the data confirm the inverse spinel structure of NiCo_2_O_4_. From this it is evident that the different composition of Ru has been successfully replaced with the Co composition. In inverse spinel patterns, half of the octahedral positions were filled by Co (Co^3+^) while the rest are occupied with Ru^34^. The intensity of diffracted peak increases with doping concentration for RNCO-4, which may be resulting from the enhanced electron density of Ru ions and lattice contraction. The well-matched characteristic peaks and the existence of Ruthenium linked impurity spikes designate the effective doping of Ru into NiCo_2_O_4_ molecule^35,36^. Figure 1b shows that low concentration of Ru doping tends to expand the lattice and promote the crystallite growth. This is likely due to the substitution effect and defect formation. The lattice constant (*a*) increased and decreased for higher concentration of doping. The increase in lattice constant is due to the incorporation of Ru^3+^ (0.68 Å) which is significantly larger than the Co^3+^ (0.545 Å). It replaces in the octahedral sites. As the larger Ru ions replace the smaller Co ions in the octahedral sites of the spinel lattice, the unit cell expands to accommodate the larger dopant, resulting in a shift of XRD peaks toward lower 2θ angles. At higher Ru-doping, the structure gets contracted and reduces crystallite size this is possible due to the increased lattice disorder, strain, or suppression of crystal growth. These changes lead to influence the physical properties such as electrical conductivity and electrochemical properties. A detailed concept of this would be discussed in the following sections.

The full width half maximum (FWHM) at (311) spike is broad, which clearly indicates the formation of nano-compounds. The following Williamson-Hall (W-H) method is used in the calculation of the crystalline properties (both strain and size) of the present nano compounds.

5 $$\beta = \beta_{{{\mathrm{cryst}}}} + \beta_{{{\mathrm{strain}}}}$$

6 $$\beta_{{{\mathrm{cryst}}}} \frac{K\lambda }{{D\cos \theta }},\;{\mathrm{and}}\;\beta_{{{\mathrm{strain}}}} = 4\varepsilon \tan \theta$$

The data is well fitted and the goodness of the plot is found to be below 2. The strain was evaluated and the results are corroborated to the structural parameters**.** A plot drawn between 4 sin $\theta$ and *β* cos $\theta$, known as W-H graph is illustrated in Fig. 1c. The line incline (K*λ/*D) explains the strain observed in the lattice while the Y-intercept gives the crystallite size (D). Here $\uplambda \left(=1.54 {\AA }\right)$ is the wave length of X-ray used in the present XRD study. The term *β* can be extracted from the FWHM obtained maximum intensity observed in the XRD data (Fig. 1b). The different slope values observed in the Fig. 1c clearly indicates the strain observed in the lattice with Ru incorporation. Moreover, the slope values were found to be positive which reveals that the strain observed in the lattice is mainly of the tensile in nature. The results were well agreed with the density location of the samples (Table 1). The decrease in δ suggests the crystal perfection and fewer defects with Ru doping. The higher Ru-doping leads to enhancement in dislocation density might be due to the formation of secondary phases and defects which increased internal micro-strain.

**Fig. 2:**
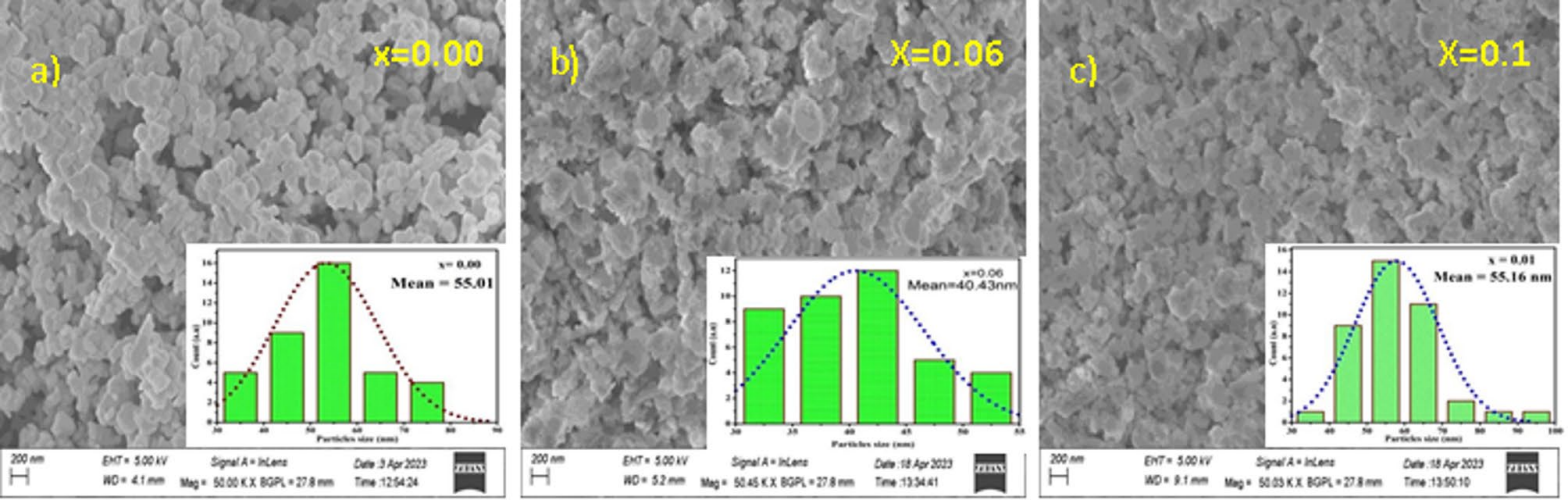
FESEM images of RNCO-1 (x = 0.0), RNCO-4 (x = 0.06), and RNCO-6 (x = 0.10).

**Fig. 3:**
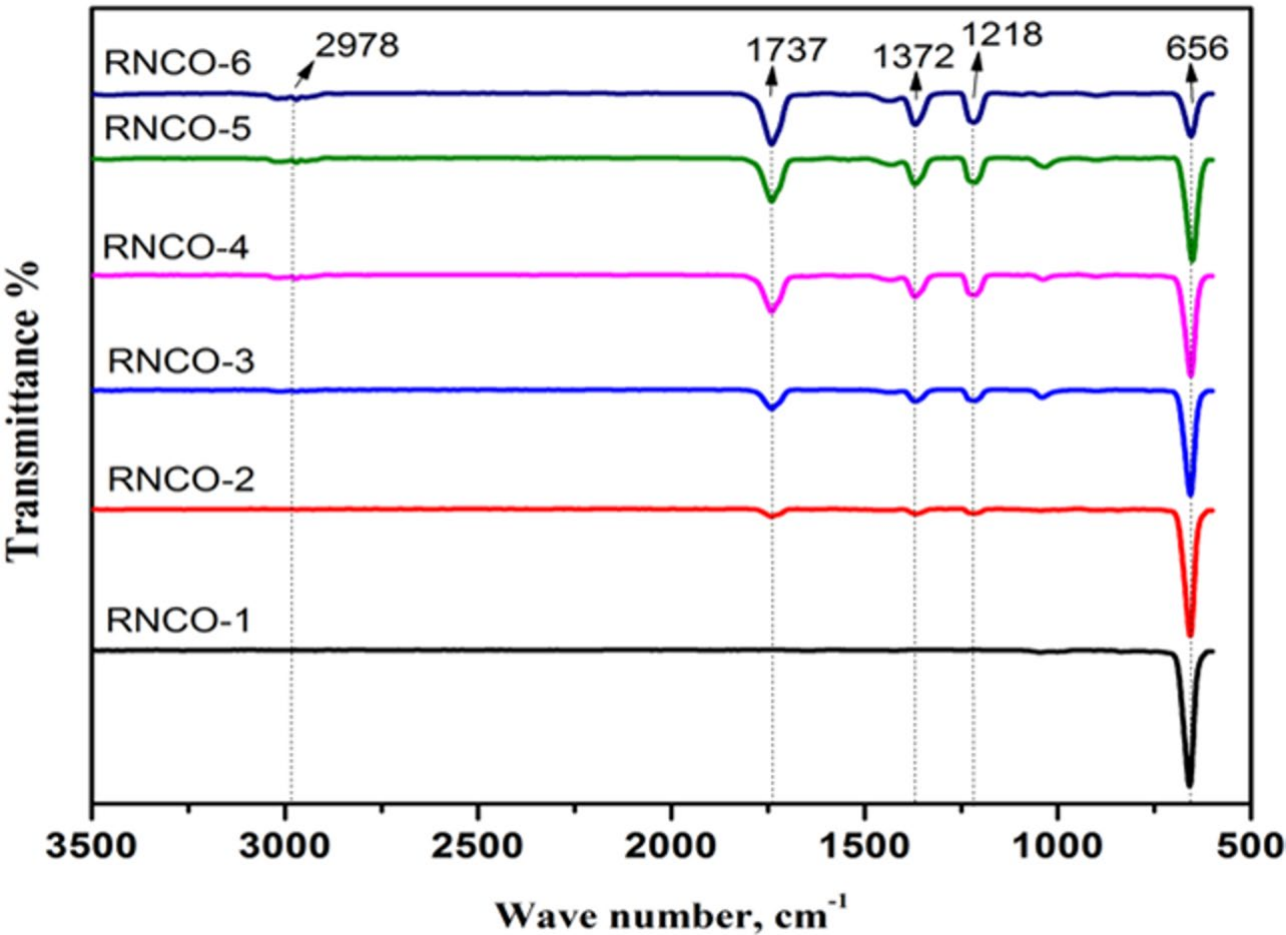
FTIR images of RNCO samples.

The surface morphology of the samples under investigation were analyzed using a FESEM (Fig. 2). The samples showed the agglomerated particle of configuration. The average grain size of RNCO-0 is 55.01 nm, RNCO-4 is having 40.43 nm and RNCO-6 has 55.16 nm. It is observed that with increase in doping concentration of the Ru, the grain size has been found to increased. This is attributed to the strain in the lattice and structural distortion^38^. The results can be visually seen in the Fig. 2. Generally, the small grain size has low activation energy, and therefore the charge transfer can take place by means of diffusion process in this type of redox reactions. The diffusion process helps the electrode material to show enhanced electrochemical performance. The band formation is analyzed in the region 500 to 3500 cm^−1^ from FTIR spectra, shown in Fig. 3. The vibration bands emerged in the vicinity of 2978, 1737, 1372, 1218, and 656 cm^−1^. The valley noticed at 2978 cm^−1^ is designated to the bending vibration of hydroxyl groups (H-OH) of the samples^42–46^. Transmittance valley observed at 1372 cm^−1^ can be assigned to the stretching vibration of residual water (H_2_O) in samples^47^, ^48^. The band observed at 1737 cm^−1^ corresponds to C=O stretching vibration mode. The valley of 656 cm^−1^ can be attributed to the Co–O vibrations from tetrahedral and octahedral sites of the present spinel structure.

**Fig. 4:**
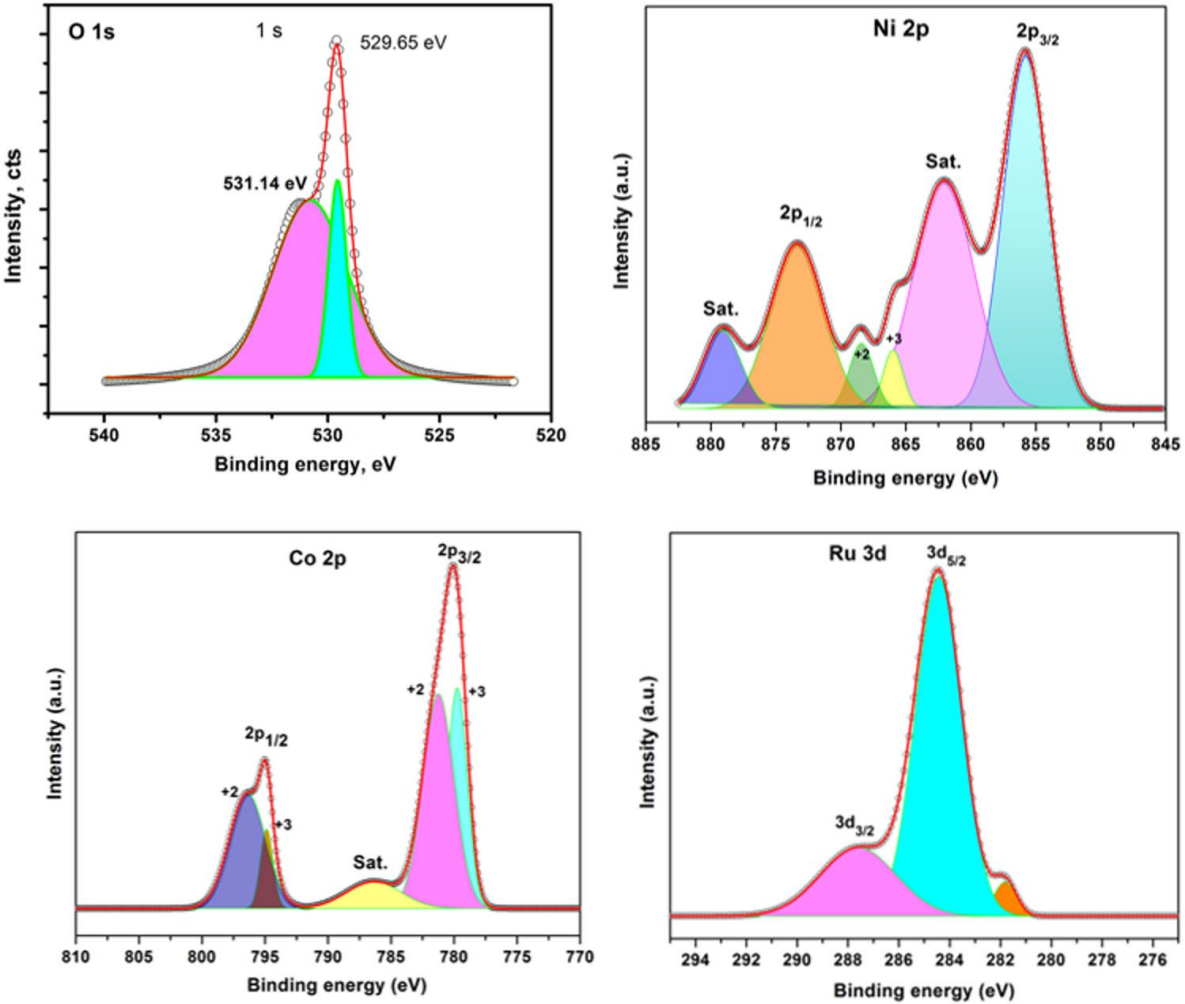
XPS spectra of RNCO-4: (**a**) O1s, (**b**) Ni 2p, (**c**) Co 2p, (**d**) Ru 3d.

The surface chemical composition and valence states of elements were detected by XPS studies. Figure 4 shows the deconvoluted XPS plot of RNCO-4 which has shown the characteristic peaks of O, Ni, Co, and Ru elements and confirms the existence of Ru, Co and Ni in the spinel. This results in creation of large Co^2+^ ions. In addition, the transformation of Co^3+^ to Co^2+^ balanced the created surface O-vacancies^49^. The O 1 s spectra is resolved into two separate peaks located at 529.65 eV and 531.14 eV. The 529.65 eV peak is related to metal oxygen bonds while 531.14 eV related to OH^−^ groups^50^.

These are clearly visible in O 1 s spectra, which are authorized to the crystal lattice (O_lat_) and adsorbed oxygen (O_ads_), respectively. The O_lat_ arises due to oxygen atoms bound to metals, and O_ads_ proposes to the highly oxidative oxygen vacancies^51^. Large magnitudes of oxygen vacancies were resulted by Ru implanting. The major difference came from the marginal composition of Ru element. Ni 2p spectra exhibited the quadruplet spikes and these are classified as Ni^2+^ and Ni^3+^ (855.37 eV, and 873.3 eV) and two shake up satellites (SS) (861.93 eV, and 878.88 eV). Co 2p contain the four peaks that are typical two peaks (780.37 eV, and 795.75 eV) and two SS peaks (786.200 eV, and 803.33 eV)^25,26^. The Ru peak found at 284 eV^52^,^53^. Plentiful oxygen voids subsist in the Ru/Ni-Co_3_O_4_ catalyst, which could reveal more functional positions and remarkably speed up the electrical conductivity and surface redox kinetics, thus amplifying the charge storage capacity.

**Fig. 5:**
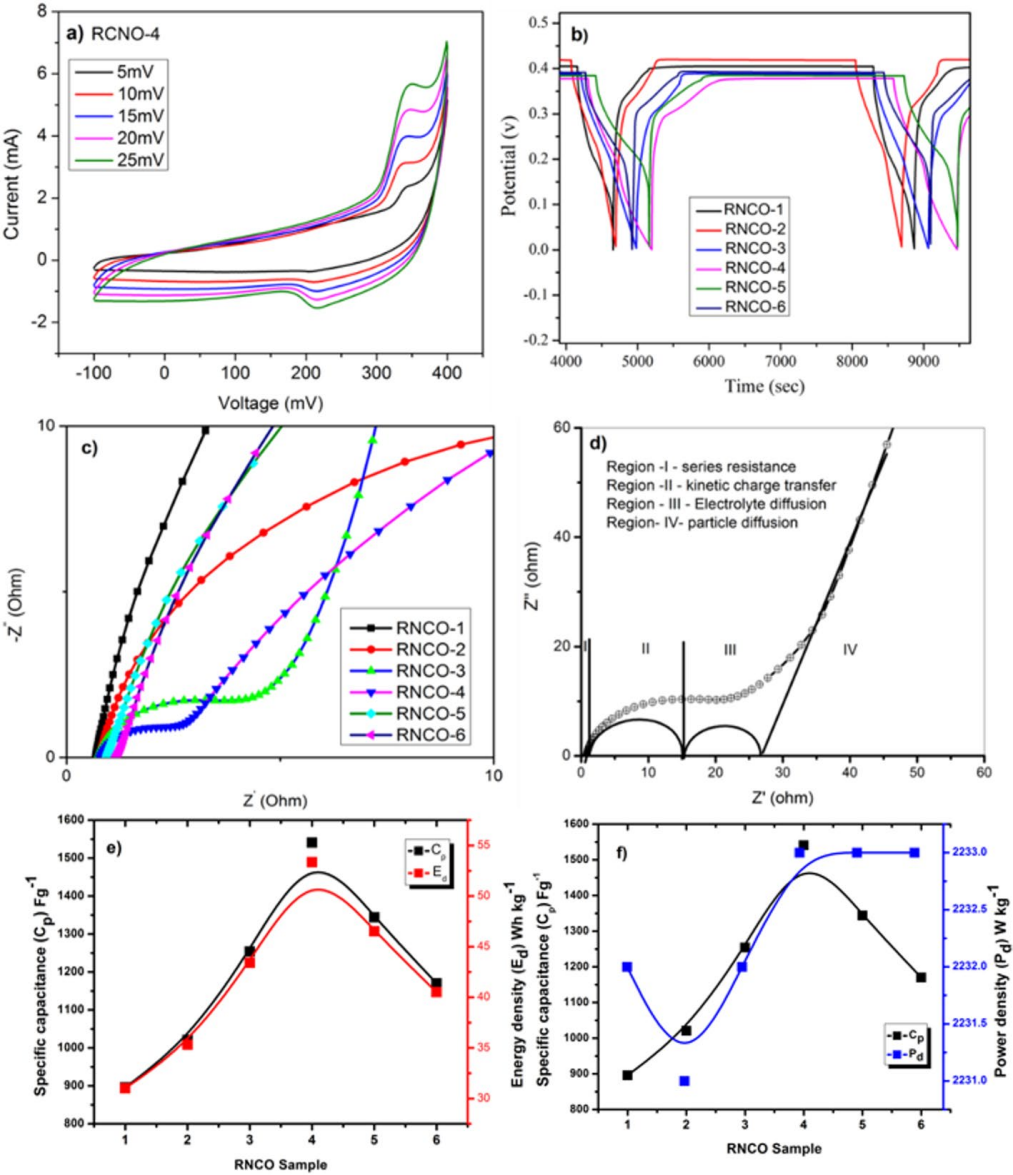
(**a**) CV of RNCO-4, (**b**) Zoomed GCD curves, and (**c**) EIS spectra, (**d**) Electro-active regions, (**e**) C_p_ and E_d _Vs. Composition (**f**) C_p_ and P_d _Vs. Composition.

The capacitive behavior of RNCO was studied using cyclic voltammetry (CV) and the results were shown in Fig. 5. The Fig. 5a depicts the response of RNCO-4. The electrodes are managed in the potential window of − 0.1 V to 0.4 V, 6.0 M KOH electrolyte and with a current 2 mA to 6 mA.  As the scan rate increases the capacitance is increased. It means the area with in the curves of CV graph is increased. When the molar ratios of Ru increases and cobalt decreases then the redox peaks positions of Ru_x_NiCo_2-x_O_4_ electrodes shifted to less positive and then negative, with increasing potential. Several pairs of typical pseudo capacitive prominent redox spikes were observed^20,46^. We can say that when the electrode has large area under low potential will gives high capacitance. Because these shifts represent the large internal resistance, which will soon hinder the ions and electrons from migrating at higher scan rates. Increased internal resistance causes the lowering of the electrochemical performance, which explains the decline of capacitance and upsurge of current density^19^.

**Fig. 6:**
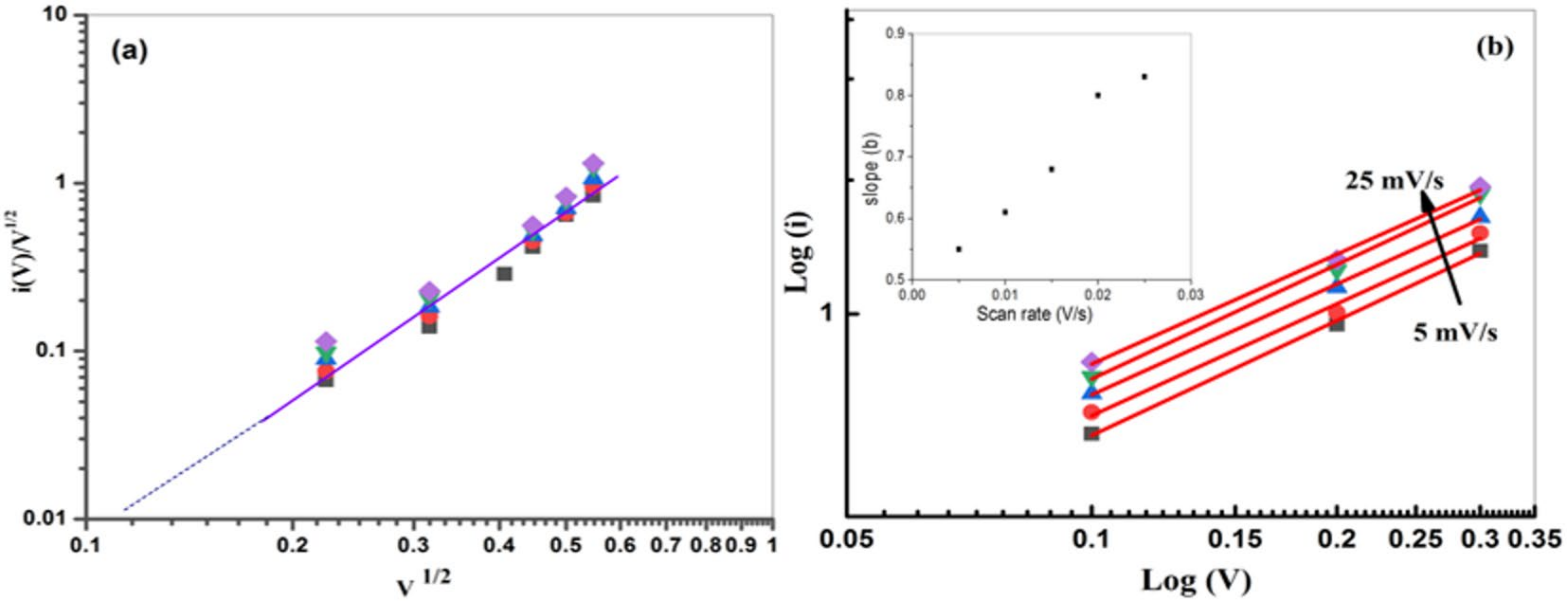
(**a**) Variation of i(V)/V^1/2^ with V^1/2^ (**b**) Log i vs Log V: (Inset) Slope-b vs Scan rate (V/s).

The Table 2 shows the specific capacitance (C_p_) of RNCO-1, RNCO-2, RNCO-3, RNCO-4, RNCO-5, and RNCO-6 were 896, 1021, 1254, 1541, 1344 and 1170 Fg^−1^ respectively. The C_p_, energy density (E_d_) at power density (P_d_) of RNCO-4 were observed to be 1541 Fg^−1^, 53.35 Wh kg^−1^ at 2233 W kg^−1^ respectively found to be better than pure NiCo_2_O_4_ (896 Fg^−1^, 31.02 Wh kg^−1^ at 2232 W kg^−1^) which resulted due to appreciably cooperative intercalation of Ni^2+^/Co^2+^ ions during redox-potential which further facilitates the ions reaching the active sites of working-electrode. The values of the table were displayed in Fig. 6e, f. The consistent globular development of NiCo_2_O_4_ nanoparticles and can help to improve the electrochemical properties like C_p_, E_d_ and P_d_ of the electrodes that exhibited the merits of doping of Ru as compared to other reports, which are found to be better placed.

**Table 2:** The magnitudes of C_p_, E_d_, and P_d_ of the Ru_x_ NiCo_2-x_O_4_ at x = 0.0–0.10.

Sl. No	Ru Concentration (x)	C_p_ Fg^−1^	E_d_ Wh kg^−1^	P_d_ W kg^−1^
1	RCNO-1	896	31.02	2232
2	RCNO-2	1021	35.33	2231
3	RCNO-3	1254	43.41	2232
4	RCNO-4	1541	53.35	2233
5	RCNO-5	1344	46.53	2233
6	RCNO-6	1170	40.51	2233

Galvano static charge/discharge (GCD) test of RNCO samples at 1 Ag^−1^ were experimented at the voltage window of 0.5 V as shown in Fig. 5b. Galvanostatic charge/discharge (GCD) plots are used to understand the underlying electrochemical energy storage activity and cycling stability. Voltage response is recorded with variation of time under constant current. The GCD curves explain the charge–discharge behavior and structural stability of a supercapacitor. The long-plateau is observed in the intermediate regions, attributed to pseudo-capacitance nature or continuous redox driven charge–discharge process of the sample. However, a drop in voltage observed in the region (> 5500 s) is attributed to the internal resistance of the sample. This process is generally observed for higher density-based electrodes. Moreover, a long plateau observed in region < 5500 s is coupled with long time discharge process. From this it is suggesting that the present materials have high energy storage capacitance nature. It should be pointed out here that the symmetric triangular regions are indication of ideal electric double layer capacitance nature of the sample. The asymmetric two triangles separated by long time indicates the EDLC behavior. All GCD curves diverge from an ideal triangular shape, confirming dominant pseudocapacitive behavior controlled by Faradaic redox reactions rather than electric double-layer capacitance. Quasi-voltage plateaus during charge/discharge indicate reversible Ni^2^^+^/Ni^3^^+^, Co^2^^+^/Co^3^^+^, and Ru-allied redox processes. Moderate Ru doping (x ≈ 0.02–0.06) yields longer discharge times, higher specific capacitance, and efficient charge storage. The small internal resistance (IR) drop reflects low IR and good conductivity. Excessive Ru slightly downgrades the supercapacitor (SC) performance. Overall, optimally Ru-doped NiCo_2_O_4_ shows high reversibility, low resistance, and excellent SC performance. It is reported that the enhanced electrochemical properties are generally attributed to high surface area of the sample. The same was not supported by the Brunauer–Emmett–Teller surface area measurements^54^. The artifact effected the total surface area of the sample by means of increasing internal pore surfaces when the material placed in the nitrogen atmosphere^55^. Many researchers have reported that the physical surface area measured in nitrogen adsorption may not directly linked to the bulky electrolyte ions instead, it depends on the conductivity, volume and the morphology of the samples. The same can be corroborated to the increased X-ray dislocation density values (see Table 1). On the other hand, in the Barrett-Joyner-Halenda (BJH) method, the calculated pore size derived from the nitrogen atmosphere and its isotherm curves which revealed that pores of 50 nm size validate the accessible ionic contributions at the surfaces. In present study, a decrease in the crystallite size certainly might have quantified the pore size distribution and allowed to enhance the dielectric values with increasing the Ru content in the lattice. From this, a tentative conclusion is arrived that the Ru substitution in the nano ferrite lattice enhances the super capacitor applications. The results were consistent with the earlier results^50^.

It is reported in the literature that the electrochemical reactions of Ru- and Co- based electrodes in the electrolyte reaction, the metal oxide or hydroxide (OH) use to involve in energy storage devices by means of electron transferred coupled reactions and maintained charge neutrality condition. The typical characteristic feature of Ru- based redox reaction and its potential effects causes the Ru- ions to form as a layer on the surfaces. These reactions can be expressed as:

7 $${\mathrm{RuO}}_{2} \; + \;{\mathrm{OH}}^{ - } \; \leftrightarrow \;{\mathrm{RuO}}_{3} ({\mathrm{OH}})\; + \;{\mathrm{e}}^{ - }$$

8 $${\mathrm{Ru}}^{3 + } ({\mathrm{surface}}) + {\mathrm{OH}}^{ - } \leftrightarrow {\mathrm{RuOH}}^{2 + } ({\mathrm{surface}})$$

The above reaction can be studied by carefully observing the cyclic voltammetry results, shown in the Fig. 5 a, b. The distinct anode peaks can be ascribed to the transition from Ru^3+^ to Ru^2+^, known as Ruthenium Redox Transition. The anodic potential dissolution of RuO_2_ generally occur in three directions, namely (i) volatilization of RuO_4_ into RuO_2_ (oxidation process) (ii) formation of soluble ruthenium-based oxide materials and (iii) detachment of RuO_2_ particles from the capacitor.

The b-value analysis (i = a V^b^) as shown Fig. 6 is insufficient to explain whether the reaction is on account of surface-controlled process or diffusion-controlled process. The exponent (b) value indicates the storage mechanism. If b = 1, then the mechanism is storage-controlled capacitive process and for b = 0.5, then the mechanism is due to diffusion-controlled process. A shift observed in the peaks position cannot quantify any particular contribution. Two methods namely Trasatti analysis and Dunn’s analysis is usually adopted. This analysis is helpful to understand the capacitive (surface-controlled) and battery (slow diffusion-controlled) methods. Surface-controlled (Dunn’s method) explains the capacitive contribution where as Transtti method deals more about the inner and outer surface charges. Higher scan rate explains capacitive contribution and lower scan rate elaborates the slower diffusion process. Both methods explain battery (slow diffusion) and supercapacitor (fast capacitive) nature. As mentioned earlier, supercapacitors exhibit both the electric double layer capacitance and pseudo-capacitance. Among various analytical methods, Dunn’s method has emerged widely employed method. The storage performance of supercapacitor occurs through two mechanisms namely, electric double layer capacitance and electromechanical (pseudo-capacitance). In double layer capacitance, the accumulation of ions may take place through electrode–electrolyte interface and it is considered as pure electrostatic mechanism (non-Faradic process), where there is no charge transfer takes place. This nature is observed in carbon-based materials. In case of pseudo-capacitor charges arrived on the rapid reversable redox reaction. This process occurs at electrode surface where charge transfer process involved. This process is called Faradic process. This nature is observed in transition metal oxides, especially in Ru_2_O based materials.

The capacitive behaviour, charge kinetics and conductivity of the electrode materials were studied by this EIS. Nyquist (Complex-impedance) plot was drawn between imaginary parts (Z”) and real part (Z’) as shown in Fig. 5c. This diagram explores information about different electro-active regions. The Nyquist plot exhibited a semi-circle in the high-frequency range and the spike in the low frequency region. The semicircle drawn at high frequency region is referred to as the interfacial charge-transfer resistance (R_ct_) formed between electrolyte–electrode interface, which can be estimated from the diameter of the semicircles outlined that the porous electrodes (RNCO-2 to RNCO-6) have a lower value of R_ct,_ compared to their solid counterparts (RNCO-1). The spike in the low frequency region is resulting from the Warburg diffusion process. The linear nature confirms the charge transfer independence of the electrode-interface^49^. The diameter of the semicircle drawn at higher frequency region (shown as solid semi-circle) explain the insulating nature of the samples. A detailed model graph is shown in Fig. 5d. From this it is evident a series resistance is added in the sample. The charge kinetic transfer takes place at higher frequency region. At Lower frequencies, the combination of both electrolyte and particle diffusion takes place. This is confirmed by observing an inclined angle (spike like behaviour) of below 45°.

From the Fig. 5d, it is observed that the smallest semicircle diameter is observed at RNCO-4, signifying reduced charge-transfer resistance and enhanced electrical conductivity. This improvement arises from easier ion transport within the electrode, promoting rapid intercalation and de-intercalation processes^50^ and resulting in superior electrochemical behaviour. The spike nature making an angle about 45° confirms the Maxwell- Wagner type as discussed above. The spike at lower frequencies suggests the thermally activated hoping electrons from the grain boundaries in the presence of slow activated applied electric fields. With increasing the frequency, the oscillating frequency restricts the tunnelling effect of electrons in the grain boundaries. From this it is concluded that the intra granular hopping mechanism is more predominant than materials as a result hopping of electrons would be combined to the restricted conductive grains interfaces.

In this paper, the complex data is fitted with Randles equivalent circuit shown in the inset Fig. 5d. The circuit explains the information about series resistance (R_s_), double layer capacitance, Warburg impedance and information about charge transfer mechanism between electrode and redox material. The Warburg impedance is related to the ion diffusion into the porous electrode. A linearly raising pattern or spike behavior observed in the low frequency region is ascribed to the ion absorption at the surface between electrode and its surface. This phenomenon is called electrode double layer formation and gives electric double layer capacitive information. This term often represented by CPE (constant phase element) of the proposed Randles equivalent circuit. The accumulated ions at the interface got diffused into the pore-based electrode of Ru. The resistance of this process is also known as charge transfer resistance (R_ct_) represented as bulk resistance, shown as first semicircle of the Fig. 5d. This term arises due to the electron transfer between the electrode and electroactive surfaces by means of adsorption or chemical binding. Showing spike of 45° or straight line in the low frequency region (region-IV) represents the diffusion of the electrocapacitive and hence mass transfer tendency dominates over the impedance nature. The second semicircle represents the pseudo-capacitance of the sample. The main aim of the supercapacitor is to store the charge and the capacity, which depends upon the ionic size and its accessibility towards the pores of the electrode. Commercially available activated carbon based electric double layer capacitors are costly and a special synthesis method is needed. Therefore, the present pseudo capacitors are prepared to store the charge by means of redox reaction. Density function theory is used to calculate quantum capacitance (QC) of supercapacitors. The quantum capacitance can be measured by the following equation:

9 $$\mathrm{Q}\mathrm{C}=\frac{dQ}{d\varnothing }$$

where $$\varnothing$$ the operating voltage and Q is the surface charge of the electrode. The specific capacitance (charge stored per unit mass or area), capacitance of electric double layer ($${C}_{EDL})$$ and supercapacitor QC are related as:

10 $$\frac{1}{C}=\frac{1}{QC}+ \frac{1}{{C}_{EDL}}$$

From the above relation it is evident that the low value of quantum capacitance decreases the electrode capacitance. The maximum energy of a supercapacitor depends on the capacitance and the operating range. The capacitance of a supercapacitor can be improved by adopting the two factors, namely (i) contribution of pseudo-Faradic, related to charge transfer process (ii) introducing transition metal oxide electrodes. The second factor improves the operating voltage and electromechanical stability of widow of electrolyte. It is reported that based on the mass ratio in hybrid supercapacitor, the maximum operating voltage can be evaluated^56^.

To fabricate asymmetric supercapacitor the charge balance should be made by considering equal and opposite charges (q+ = q−). The charge stored in the electrode depends on the specific capacitance, potential of charge/discharge ($\Delta$V) and mass of the electrode. Therefore, the mass balance is made by using the following relation:

11 $$\frac{{m}^{+}}{{m}^{-} }=\frac{({C}^{-} \times {\Delta \mathrm{V}}^{-})}{({C}^{+} \times {\Delta \mathrm{V}}^{+}.}$$

As mentioned earlier the spike observed in the Cole–Cole plot is attributed to the different charge storage and slow conductivity mechanism occur at low frequency domain. The impendence is also bound to decrease with the Ru—composition. The resistance of RCNO-4 is very small compared to the other samples. Ragone plot values were found to be important for high-performance supercapacitors applications. The values are strictly checked with the standard windows and electrolyte decomposition limits in order to ensure practical applications^49,50^.

## Conclusion

The Ru_x_NiCo_2-x_O_4_ powders were synthesized using microwave hydrothermal (M-H) and fabricated electrode on Ni foam. These samples exhibit an inverse spinel structure with half of the Co and Ni ions occupy octahedral sites, where Ru substitutes at Co positions. This study demonstrates the advantages of metal doping achieved through the M-H synthesis method and highlights its potential for developing advanced SC electrode materials. The spike observed at lower frequency is attributed to the Ru-ions in the electrolyte have sufficient time to mix into the electrode surface and form as a stable double layer capacitor. This is evident from the SEM picture by exhibiting non-uniform pore size distribution.

A superior specific capacitance and energy density for RCNO-4 is found to be 1541 Fg^−1^ with 53.35 Wh kg^−1^ and based on this value, it is tentatively concluded that this material is found to be future promising SC applications. The result was also corroborated to a model proposed in the complex impedance analysis. This trend is also confirmed by observing by two slopes in W-H plot. It is being considered in the literature that the low impedance is a necessary condition for a super capacitive behavior. But this is not a sufficient condition for better performance. However further results are needed to confirm the same. More studies on nitrogen environment help to understand the electronic coupling mechanism for the development of sensor.

## Experimental

### Materials used

All the chemical reagents of high purity were used without any further purification. The reactants were; Ruthenium chloride hexahydrate (RuCl_3_.3H_2_O), nickel nitrate hexahydrate (Ni(No_3_)_2_.6H_2_O), cobalt nitrate hexahydrate (Co(NO_3_)_2_.6H_2_O), sodium hydroxide (NaOH) pellets, potassium hydroxide (KOH), pure ethanol and polyvinyl alcohol, Deionized water were used to dissolve the reactants.

### Synthesis of RNCO

**Fig. 7:**
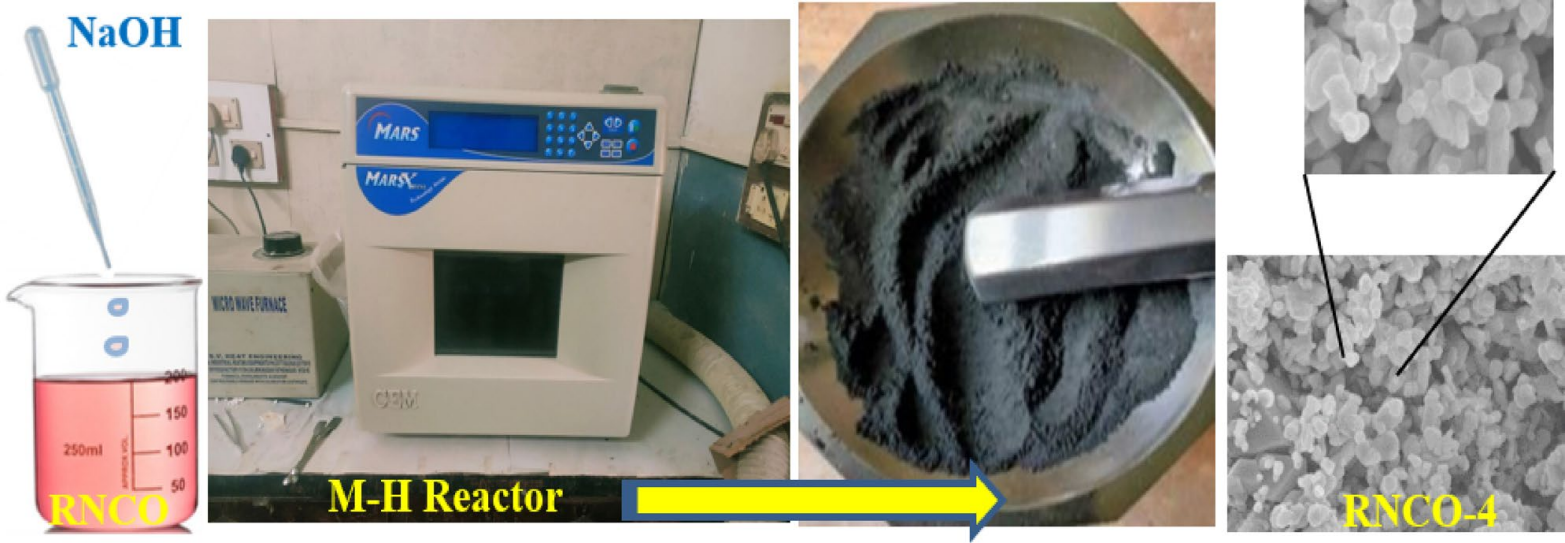
Schematic representation of preparing Ru_x_NiCo_2-x_O_4_ simple one-step M-H treatment as efficient electrode materials for charge storage.

The Ru doped with NiCo_2_O_4_ nanoparticles with large number of vacancy defects were prepared using microwave hydrothermal (M-H) synthesis followed by slight co-precipitation as shown in the schematic diagram of Fig. 7. All the laboratory equipment used for synthesis were cleaned thoroughly. To synthesis the Ruthenium Nickel Cobaltite, Ru_x_NiCo_2-x_O_4_ for x = 0.0, 0.02, 0.04, 0.06, 0.08, and 0.10, the Cobalt is replaced with Ru composition according to the stoichiometric ratios. Initially, Co(NO_3_)_2_.6H_2_O (cobalt nitrate hexa hydrate), and RuCl_3_.3H_2_O (Ruthenium chloride trihydrate) were completely dissolved in the 40 ml DI water (deionized water) under uniform stirring for 30 min with Co/Ru ratios of 100:0, 98:2, 96:4, 94:6, 92:8, 90:10. Then, added Ni(No_3_)_2_.6H_2_O to the above solution, and stirred again for 30 min. Few drops of (NaOH) was added to the above mixture to maintain a pH of 11. While stirring the solution with NaOH, an exothermic reaction takes place forming semi-gel like solution. The resulting mixture was transferred into Teflon tubes then sealed tightly and placed in the microwave reactor which is maintained at a temperature of 160 °C for 45 min. Later it is allowed to cool for an hour. The precipitation from Teflon tubes transferred in to their respective beakers and carried out co-precipitation method to wash the mixtures for about 15–20 washes. Subsequently, the washed precipitate of the respective beakers kept in an oven for 12 h at 70 °C. The dried-out mixture is then grinded for an hour into fine particles using mortar and pestle. The powder is then sintered at 900 °C for 4 h. The sintered powders were grounded for 6 h and labeled as RNCO-1, RNCO-2, RNCO-3, RNCO-4, RNCO-5, RNCO-6. The electrochemical study was made for the samples using the CV.

### Electrode fabrication

Keeping in view of above relation**, t**he homogenous slurry of prepared RNCO nano powder, CB (carbon) black, and PVDF (polyvinylidene fluoride) in a weight percentage of 80:10:10 were mixed using N-methyl-2-pyrrolidone (C_5_H_9_NO) to prepare a paste (thick slurry). The Ni foam cut into 1 × 2 cm and was cleaned with acetone, 1 × 1 cm pieces were coated with the prepared slurry, allowed them to dry in the hot air oven for 6 h at 80 °C which is used as the working electrode for studying the electrochemical properties.

### Material characterization

The XRD (X-ray diffraction) patterns were obtained using Shimadzu-XRD#7000 instrument used with Cu-K_α_ radiation of wave length of1.5405 Å, the crystalline phases and structure of the samples were examined. The field emission scanning electron microscope (FESEM) (FEI ApreoS and OXFORD X-Max) fitted with an energy dispersive X-ray detector (EDAX, 15 kV) is used to investigate the topology, morphology and elemental compositions of the prepared materials. The Fourier transform infrared spectrometer (FTIR) (Shimadzu, 8400 S) to characterize the structural details and functional groups of the produced samples within 500–3500 cm^–1^ wave number range. The elemental composition, chemical and electronic state of the atoms within the material was characterized by XPS (X-ray photo-electron spectroscopy) using Kratos-Axis Ultra DLD instrument.

### Electrochemical measurements

Prepared electrode samples were electrochemically analyzed at room temperature using CV, GCD, and EIS to investigate the electrochemical properties. A three-electrode system was used in this experiment for the measurement of the prepared slurry samples coated on 1 × 1 cm^2^ Ni foam which is used as working-electrode, a platinum wire as counter- electrode, and an Ag/AgCl as reference-electrode. An aqueous-electrolyte solution of 6 M KOH was used to carry out the electrochemical measurements. The CV studies are carried out at a potential window of 0–1 V with varying scan rates of potential 5, 10, 20, 50, and 100 mVs^−1^. At their open circuit potentials, the EIS studies were carried out in the frequency range 0.1 to 10^5^ Hz and the specific capacitance (C_p_), the E_d_ and P_d_ densities were measured by GCD characterization studies^53^.

## Supplementary Information

Below is the link to the electronic supplementary material.

Supplementary Material 1

## Data Availability

Data supporting the findings of this study are available from the corresponding author upon reasonable request.

## References

[1] Salanne, M. et al. Efficient storage mechanisms for building better supercapacitors. *Nat. Energy* **1**, 16070. 10.1038/nenergy.2016.70 (2016).

[2] Wei, J. S. et al. Robust negative electrode materials derived from carbon dots and porous hydrogel for high-performance hybrid supercapacitors. *Adv. Mater.* **31**, 1806197. 10.1002/adma.201806197 (2019). 10.1002/adma.201806197 30537033

[3] Zhong, C. et al. A review of electrolyte materials and compositions for electrochemical supercapacitors. *Chem. Soc. Rev.* **44**, 7484–7539 (2015). 26050756 10.1039/c5cs00303b

[4] Xiao, J. W., Wan, L., Yang, S. H., Xiao, F. & Wang, S. Hierarchical NiCo_2_S_4_ nanotube arrays on carbon fiber paper for high-performance pseudocapacitors. *Nano Lett.* **14**, 831–838 (2014). 24437988 10.1021/nl404199v

[5] Jiang, H., Lee, P. S. & Li, C. Z. 3D carbon-based nanostructures for advanced supercapacitors. *Energy Environ. Sci.* **6**, 41–53 (2013).

[6] Miller, J. R. & Simon, P. Electrochemical capacitors for energy management. *Science* **321**, 651–652 (2008). 18669852 10.1126/science.1158736

[7] Xin, H., Xu, Z., Liu, Y., Li, W. & Hu, Z. 3D flower-like Ni_2_Co_2_O_4_ electrode material prepared by a modified solvothermal method for supercapacitors. *J. Alloy. Compd.*10.1016/j.jallcom.2017.03.208 (2017).

[8] Wei, T. Y., Chen, C. H., Chien, H. C., Lu, S. Y. & Hu, C. C. Spinel nickel cobaltite aerogels as cost-effective supercapacitor materials. *Adv. Mater.* **22**, 347–351 (2010). 20217716 10.1002/adma.200902175

[9] Liew, C.-W., Ramesh, S. & Arof, A. K. Enhanced capacitance of EDLCs (electrical double layer capacitors) based on ionic liquid-added polymer electrolytes. *Energy* **109**, 546–556 (2026).

[10] Bharti, et al. Theories and models of supercapacitors with recent advancements: impact and interpretations. *Nano Express* **2**, 022004 (2021).

[11] Peng, C. et al. Regulating cobalt valence in NiCo_2_O_4_ via Ru substitution for enhanced oxygen evolution reaction. *Appl. Surf. Sci.* **544**, 148897 (2021).

[12] Liu, S., Hui, K. S. & Hui, K. N. Flower-like copper cobaltite nanosheets on graphite paper for supercapacitors and glucose sensing. *ACS Appl. Mater. Interfaces* **8**, 3258–3267 (2016). 26757795 10.1021/acsami.5b11001

[13] Krishnan, S. G. et al. MgCo_2_O_4_ as an electrode for high-performance supercapacitors. *Electrochim. Acta* **161**, 312–321 (2015).

[14] Kadam, R. A. et al. Bimetallic Co_3_V_2_O_8_ microstructure: A versatile bifunctional electrode for supercapacitor and electrocatalysis applications. *Surf. Interfaces* **41**, 1032676 (2023).

[15] Tomy, M., Ambika Rajappan, A., Vm, V. & Thankappan Suryabai, X. Emergence of novel 2D materials for high-performance supercapacitor electrode applications: a brief review. *Energy Fuels* **35**, 19881–19900 (2021).

[16] Teli, A. M. et al. Kim HH Innovations in metal telluride composite materials towards enhancing supercapacitor energy storage. *J. Alloys Compounds* **1005**, 175950 (2024).

[17] Leea, S. C. & Jungb, W. Y. Analogical understanding of the Ragone plot and a new categorization of energy devices. *Energy Procedia* **88**, 526–530 (2016).

[18] Silambarasan, M., Ramesh, P. S. & Geetha, D. One-step synthesis and electrochemical properties of NiCo_2_O_4_ nanostructures. *J. Mater. Sci. Mater. Electron.* **27**, 11145–11154. 10.1007/s10854-016-5527-9 (2016).

[19] Yang, S., Yu, J., Jiang, T. T., Zhu, L. X. & Xu, X. L. Ru_x_Ni_1−x_Co_2_O_4_ grown on nickel foam for solid-state supercapacitors. *J. Alloys Compd.* **764**, 767–775 (2018).

[20] Qiu, K. W. et al. ZnCo_2_O_4_/MnO_2_ nanocone forests for high-performance supercapacitors. *Nano Energy* **11**, 687–696 (2015).

[21] Jadhav, H. S., Roy, A., Chung, W. J. & Seo, J. G. Urchin-like ZnCo_2_O_4_ microspheres on nickel foam for supercapacitors. *Electrochim. Acta* **246**, 941–950 (2017).

[22] Pendashteh, A. et al. Mesoporous CuCo_2_O_4_ nanowires for supercapacitors. *Chem. Mater.* **27**, 3919–3926 (2015).

[23] Vijayakumar, S., Nagamuthu, S. & Ryu, K. S. CuCo_2_O_4_ flowers on Ni foam for hybrid supercapacitors. *Electrochim. Acta* **238**, 99–106 (2017).

[24] Dong, Y. et al. Hierarchical MnCo_2_O_4_ nanocages for supercapacitors. *Electrochim. Acta* **225**, 39–46 (2017).

[25] Huang, T. F. et al. Porous MnCo_2_O_4_ via combustion synthesis for supercapacitors. *Ceram. Int.* **43**, 1968–1974 (2017).

[26] Sun, S. et al. NiCo_2_O_4_/3D graphene based asymmetric supercapacitors. *J. Mater. Chem. A* **4**, 18646–18653 (2016).

[27] Gao, G. X., Wu, H. B., Ding, S. J., Liu, L. M. & Lou, X. W. Hierarchical NiCo_2_O_4_ nanosheets on Ni nanofoam. *Small* **11**, 804–808 (2015). 25228205 10.1002/smll.201402539

[28] Zhang, Y. Y. et al. Ni@NiCo_2_O_4_ core–shell composites for supercapacitors. *Ceram. Int.* **43**, 2057–2062 (2017).

[29] Zheng, C. R., Cao, C. B., Chang, R. L., Hou, J. H. & Zhai, H. Z. Mesoporous NiCo_2_O_4_ hollow nanocubes for supercapacitors. *Phys. Chem. Chem. Phys.* **18**, 6268–6274. 10.1039/C5CP07997G (2016). 26853189 10.1039/c5cp07997g

[30] Vidhya, M. S. et al. Nickel–cobalt hydroxide as a positive electrode for supercapacitors. *RSC Adv.* **10**, 19410–19418 (2020). 35515465 10.1039/d0ra01890b PMC9054063

[31] Koneti, B. B., Chidurala, S. C., Katlakunta, S., Thida, R. K. & Butreddy, R. R. Al-doped nickel cobaltite nanoparticles synthesized by microwave hydrothermal method. *High Technol. Lett.* **30**, 145–164 (2024).

[32] Elessawy, N. A., El Nady, J., Wazeer, W. & Kashyout, A. B. Nitrogen-doped graphene for high-performance supercapacitors. *Sci. Rep.* **9**, 1129 (2019). 30718552 10.1038/s41598-018-37369-x PMC6362120

[33] Yu, M. et al. Mesoporous NiCo_2_O_4_ nanoneedles on graphene–nickel foam for supercapacitors. *Electrochim. Acta* **165**, 99–108 (2015).

[34] Che, H. et al. Flower-like Ni_x_Co_3−x_O_4_ microstructures for supercapacitors. *Electrochim. Acta* **247**, 283–291 (2017).

[35] Xie, Q. et al. Core-shell N-doped carbon fiber@graphene composites for aqueous symmetric supercapacitors with high-energy and high-power density. *J. Power. Sources.* **317**, 133–142 (2016).

[36] Dubal, D. P., Chodankar, N. R., Holze, R., Kim, D. H. & Gomez-Romero, P. Ultrathin mesoporous RuCo_2_O_4_ nanoflakes for asymmetric supercapacitors. *Chem. Sus. Chem.*10.1002/cssc.201700001 (2017). 10.1002/cssc.201700001 28158923

[37] Wang, P. F. et al. Carbon/Carbon supported RuO2 nanoparticles with a hollow interior s excellent material for supercapacitors. *Nano Energy*. **5**, 116–124. 10.1016/j.nanoen.2015.04.006 (2015)

[38] Yewale, A. B. S. H. M., Akkinepally, B. & Shin, D. K. Green batteries: A sustainable approach towards next-generation batteries. *Batteries* **11**(7), 258. 10.3390/batteries11070258 (2025).

[39] Morankar, P. J. et al. Surfactant-directed hydrothermal synthesis of 3D niobium phosphate micro-flowers for enhanced asymmetric supercapacitors. *J. Power Sources*10.1016/j.jpowsour.2025.236813 (2025).

[40] Hao, J. et al. Effect of grain size on electrochemical performance of Co_3_O_4_. *J. Mater. Chem. A* **8**, 7192–7196 (2020).

[41] Dubal, D. P., Gund, G. S., Lokhande, C. D. & Holze, R. CuO cauliflower structures for supercapacitors. *Mater. Res. Bull.* **48**, 923–928 (2013).

[42] Liu, S., Hu, L., Xu, X., Al-Ghamdi, A. A. & Fang, X. Nickel cobaltite nanostructures for photoelectric and catalytic applications. *Small* **11**, 4267–4283 (2015). 26121217 10.1002/smll.201500315

[43] Zhang, G. Q. & Lou, X. W. Mesoporous NiCo_2_O_4_ nanosheets for supercapacitors. *Adv. Mater.* **25**, 976–979 (2013). 23225205 10.1002/adma.201204128

[44] Wu, C. et al. Mesoporous Zn–Ni–Co oxide nanowires on nickel foam. *ACS Appl. Mater. Interfaces* **7**, 26512–26521 (2015). 26575957 10.1021/acsami.5b07607

[45] Liu, H. B., Xiang, L. & Jin, Y. Hydrothermal modification of Ni(OH)_2_ with high discharge capability. *Cryst. Growth Des.* **6**, 283–286 (2006).

[46] Yan, D. et al. Biomorphic Co₃O_4_ microtubules for pseudocapacitors. *ACS Appl. Mater. Interfaces* **6**, 15632–15637 (2014). 25207997 10.1021/am5044449

[47] Sathyanarayana, N., Kumar, R., Shilpa Chakra, T., Sreenivasu, C. & Ravinder Reddy, D. Ca-doped NiCo_2_O_4_ nanoparticles synthesized by microwave hydrothermal method. *Int. J. Eng. Res. Appl.* **13**, 29–41 (2023).

[48] Yuan, C. Z. et al. Ultrathin mesoporous NiCo_2_O_4_ nanosheets on Ni foam. *Adv. Funct. Mater.* **22**, 4592–4597 (2012).

[49] Saveleva, V. A. et al. Surface chemistry of transition-metal oxides. *J. Phys. Chem. Lett.* **7**, 3240–3245 (2016). 27477824 10.1021/acs.jpclett.6b01500

[50] Atanasoska, L., Rady, W. G., Atanasoski, R. T. & Pollak, F. Surface electronic properties of oxides. *Surf. Sci.* **202**, 142–166 (1988).

[51] Toghan, A., Khairy, M., Kamar, E. M. & Mousa, M. A. Effect of particle size on NiFe_2_O_4_ for supercapacitors. *J. Mater. Res. Technol.* **19**, 3521–3533 (2022).

[52] Li, Y., Han, X., Yi, T., He, Y. & Li, X. Review of NiCo_2_O_4_-based composites for supercapacitors. *J. Energy Chem.* **31**, 54–78 (2019).

[53] Sathyanarayana, M. et al. Zn-doped NiCo_2_O_4_ synthesized by microwave hydrothermal process. *ECS J. Solid State Sci. Technol.* **12**, 093008 (2023).

[54] Yewale, M. A. & Shin, D. K. Improvement of Co_3_V_2_O_8_ nanowire driven by morphology for supercapacitor and water splitting applications. *Batteries* **11**(4), 118. 10.3390/batteries11040118 (2025).

[55] Morankar, P. J. et al. Asymmetric supercapacitor performance enhancement through Fe-doped MoS_2_ nanosheets synthesized via hydrothermal method. *Coatings* **14**, 1328. 10.3390/coatings14101328 (2024).

[56] Pawar, C. A., Sharma, A. K., Prasad, N. R., Suryawanshi, S. S. & Yewale, M. A. *Murraya koenigii* assisted synthesis of bioactive silver nanomaterial. *Macromol. Symp.*10.1002/masy.202100126 (2021).

