# Supplementary figures and images for: Microwave-hydrothermally synthesized RuxNiCo2-xO4 spinel oxide nanoparticles for high-performance pseudocapacitor electrodes
Source: Sci Rep. 2026 May 12;16:22655. doi: 10.1038/s41598-026-52710-5 (PMC13385366; doi:10.1038/s41598-026-52710-5)

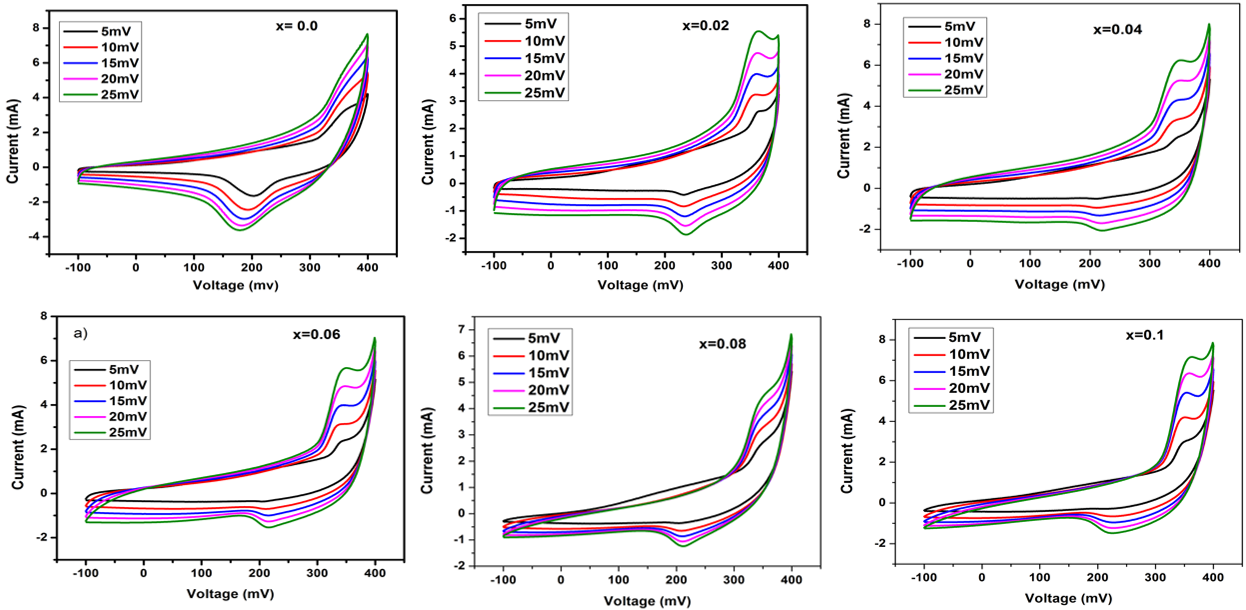


**Figure S1:** CV plots of RNCO at scan rates of 5, 10, 15, 20, 25 mV/s

Supplement: Supplementary file 1 — Supplementary Material 1 [file 41598_2026_52710_MOESM1_ESM.docx]
